# Management of ectopic pregnancy after in vitro fertilization/intracytoplasmic sperm injection and embryo transfer: a case series and mini-review

**DOI:** 10.2478/abm-2024-0004

**Published:** 2024-03-20

**Authors:** Yang Zhang, Yan Zhai, Danni Qu

**Affiliations:** Medical Center for Human Reproduction, Beijing Chao-Yang Hospital, Capital Medical University, Beijing 100020, China; Department of Obstetrics and Gynecology, Beijing Chao-Yang Hospital, Capital Medical University, Beijing 100020, China

**Keywords:** assisted reproductive technology, ectopic pregnancy, embryo transfer, intracytoplasmic sperm injection, in vitro fertilization

## Abstract

**Background:**

Ectopic pregnancy (EP), reflecting a fertilized ovum implanted outside the normal uterine cavity, represents a frequent cause of morbidity and possibly mortality in women of reproductive age.

**Objective:**

To summarize the diagnosis and treatment of EP after in vitro fertilization/intracytoplasmic sperm injection and embryo transfer (IVF/ICSI-ET).

**Methods:**

The medical records of patients who were diagnosed with EP after embryo transfer from 2017 to 2019, in a tertiary hospital were reviewed.

**Results:**

Of the 24 cases analyzed, 21 (87.5%) had fallopian tube involvement, while 2 (8.3%) and 1 (4.2%) had cornual and cervical pregnancies, respectively. Clinical manifestations included vaginal bleeding (58.3%) and lower abdominal pain (16.7%); 9 (42.9%) cases had no symptoms. One cornual pregnancy was misdiagnosed as acute appendicitis and later correctly diagnosed by laparoscopic exploration. There were 2 cases of multiple-site EP and 2 of heterotopic pregnancy, including one with an intrauterine pregnancy with double chorionic and four amniotic sacs and right tubal ampullary pregnancy. Five of the 21 cases with fallopian tube involvement received conservative treatment, while the remaining 16 underwent surgeries, including laparoscopic ipsilateral salpingostomy and ipsilateral salpingectomy.

**Discussion:**

Ectopic pregnancy after embryo transfer, mainly involving the fallopian tube, is very complex and is with diverse manifestations. Even with the pregnancy sac observed in the uterus, the pelvic cavity should be scanned thoroughly after embryo transfer.

Ectopic pregnancy (EP), reflecting a fertilized ovum implanted outside the normal uterine cavity, represents a frequent cause of morbidity and possibly mortality in women of reproductive age [1]. Indeed, EP is the most common cause of maternal death in the first trimester of pregnancy. The incidence of EP after natural conception is about 1%–2% and most commonly involves the fallopian tube, which accounts for almost 95% cases [2]. A previous history of EP may cause infertility; meanwhile, the incidence of EP may be increased after assisted reproductive technology (ART) treatments, including intrauterine insemination, in vitro fertilization or intracytoplasmic sperm injection and embryo transfer (IVF/ICSI-ET), to about 1.4%–5.4% [3]. This indicates a complex association between infertility and EP, as one of these ailments could be simultaneously a cause and the consequence of the other [4].

Currently, infertility has become a major health issue, affecting 8%–12% of couples worldwide [5]. With the current advances in ART, this treatment is increasingly being requested. Consequently, EP after ART is gaining increased attention. Risk factors for EP after IVF/ICSI-ET are associated with the infertility history of the patient or specific parameters related to the applied IVF/ICSI-ET technique; therefore, a model has been proposed to group the women undergoing ART based on the incidence of EP for reducing the medical resources spent on individuals with low-risk EP and for providing targeted, tailored treatment to those at a higher risk of EP [6].

To contribute to the knowledge related to EP after ART, this study aimed to retrospectively analyze the cases of EP secondary to IVF/ICSI-ET from a tertiary hospital, highlighting diagnosis and treatment.

## Methods

All cases of EP after embryo transfer, admitted to the Department of Gynecology of Beijing Chao-Yang Hospital at the Capital Medical University from January 1, 2017 to December 31, 2019, were enrolled. The inclusion criteria were: (1) pregnancy after embryo transfer and (2) clinical (ultrasound) or surgical diagnosis of EP. The exclusion criteria were: (1) EP after natural conception and (2) intrauterine pregnancy after embryo transfer. This study was approved by the ethics committees of Beijing Chaoyang Hospital affiliated to Beijing Capital Medical University (2020-Research-304). This article was a retrospective study. Therefore, the requirement for written informed consent from the patients was waived. All patients received assisted reproductive therapy for infertility: superovulation, egg retrieval, embryo transfer (fresh cycle or frozen cycle), and luteal support. Pregnancy was confirmed biochemically by serum hCG (human chorionic gonadotropin)>15 IU/L after 14 days of embryo transfer. Clinical pregnancy was determined by observing the gestational sac and primitive cardiac pulsation by ultrasound at 28 days after embryo transfer. Patient data were collected, including age, type of embryo transfer (fresh cycle or frozen cycle), type of embryo transferred, average duration from embryo transfer to the diagnosis of EP, site of EP, clinical manifestations, and treatment methods. Data analysis was performed with the IBM SPSS21.0 software (IBM). Categorical variables were expressed as rate (%).

## Results

Concerning the inclusion criteria of this study, we recruited 24 patients who were diagnosed with EP after embryo transfer. Patient demographics and basic characteristics are summarized in **Table 1**. The average age of patients was 32.5 years, ranging from 24 years to 40 years. The average time from embryo transfer to the diagnosis of EP was 27.2 days, ranging from 19 days to 59 days. The incidence of EP after embryo transfer in the reproductive center from January 1, 2017 to December 31, 2019 was 1.31% (33/2510). Of the 24 cases analyzed in this study, 21 (87.5%) had fallopian tube involvement, while 2 (8.3%) and 1 (4.2%) had cornual and cervical pregnancies, respectively. Clinical manifestations included vaginal bleeding in 14 (58.3%) cases and lower abdominal pain in 4 (16.7%). One (4.8%) patient presented only abdominal pain. There were 9 (42.9%) asymptomatic cases. In total, 18 patients received frozen cycle embryo transfer and 4 received fresh cycle. The remaining two patients received embryo transfer in other hospitals, with no medical records available. Of the types of embryos transferred, 5 cases were at the blastocyst stage, 3 cases were at the cleavage stage, and the rest included both. Totally, 17 (85%) patients were transferred with 2 embryos, 3 patients with 1 embryo, and 4 patients had no medical records available.

**Table 1.** Cases of ectopic pregnancy following embryo transfer

**Case**	**Age (years)**	**Number of transferred embryos**	**Days after embryo transfer**	**Clinical manifestations**		**Diagnosis**	**Treatment**
				**Abdominal pain**	**Vaginal bleeding**		
1	36	2	33	−	+	Intrauterine pregnancy with right tubal ampullary pregnancy	Right side salpingectomy
2	24	NA	21	+	+	Intrauterine pregnancy with right tubal ampullary pregnancy	Right side salpingectomy + Fetal reduction
3	32	2	26	−	−	Right ampullary pregnancy with right isthmus pregnancy	Right side salpingectomy
4	29	2	26	+	+	Right ampullary pregnancy with ruptured right interstitial pregnancy	Both side salpingectomy
5	29	2	26	−	−	Left tubal ampullary pregnancy and right hydrosalpinx	Both side salpingectomy
6	29	NA	37	+	+	Right tubal ampullary pregnancy	Right side salpingostomy
7	31	2	37	+	+	Right tubal ampullary pregnancy	Right side salpingostomy + methotrexate
8	40	2	28	−	+	Right tubal ampullary pregnancy	Right side salpingectomy
9	33	2	19	−	+	Right tubal ampullary pregnancy	Both side salpingectomy
10	29	2	20	−	+	Right tubal ampullary pregnancy	Left side salpingectomy + Curettage
11	30	1	22	−	−	Right tubal ampullary pregnancy	Both side salpingectomy
12	30	1	28	−	+	Right tubal ampullary pregnancy	Right side salpingectomy
13	28	2	27	−	+	Left tubal ampullary pregnancy	Left side salpingectomy
14	35	2	26	−	−	Right tubal interstitial pregnancy	Right side salpingectomy + methotrexate
15	36	2	28	−	−	Right tubal interstitial pregnancy	Right side salpingectomy
16	39	NA	25	−	+	Left tubal interstitial pregnancy	Left side salpingectomy
17	33	2	30	−	−	Left ectopic pregnancy	Conservative treatment
18	35	2	23	−	+	Right ectopic pregnancy	Conservative treatment
19	30	2	24	−	+	Left ectopic pregnancy	Conservative treatment
20	30	1	22	−	−	Right ectopic pregnancy	Conservative treatment + Mifepristone
21	28	2	20	+	+	Right ectopic pregnancy	Conservative treatment
22	39	2	22	−	−	Right cornual pregnancy	Curettage
23	34	2	24	+	−	Right cornual pregnancy	Uterus repair
24	40	NA	59	−	−	Cervical pregnancy	Clamping

In the 16 tubal pregnancy cases who underwent surgery, the ampullary region of the fallopian tube was the most common site of implantation (13/16, 81.3%). There were 2 cases of heterotopic pregnancy. Case 1 had intrauterine pregnancy with right tubal ampullary pregnancy. Ultrasound only showed a single fetal sac on the 19th day after embryo transfer, suggesting intrauterine pregnancy for 4 weeks, accompanied by little vaginal bleeding. On the 26th day after embryo transfer, ultrasound faintly showed a punctate fetal bud and fetal heartbeat. On the 33th day after embryo transfer, ultrasound confirmed the intrauterine pregnancy in addition to the right EP. The patient underwent emergency treatment by laparoscopic right salpingectomy. The intrauterine pregnancy in the patient lasted to full term, and a healthy baby was delivered. Case 2 had sexual intercourse before embryo transfer. She suffered from vaginal bleeding with right lower quadrant pain on the 21st day after the embryo transfer. Ultrasound showed intrauterine pregnancy with double chorionic and four amniotic sacs, with abnormal echo in the right attachment area that prompted EP. Laparoscopic surgery was performed on the day of diagnosis and the right ampullary pregnancy was observed perioperatively; therefore, right fallopian tube resection was performed. After the operation, two fetuses were removed and the other two were preserved. There were two cases of multiple site EP. Case 3 had ampullary pregnancy with ipsilateral isthmus pregnancy; case 4 had ampullary pregnancy with ruptured ipsilateral interstitial pregnancy. Both case 3 and 4 underwent salpingectomy. Five of the 21 tubal EP cases received conservative treatments. Sixteen of the 21 underwent surgical treatments, including laparoscopic ipsilateral salpingostomy (*n* = 2) and ipsilateral salpingectomy (*n* = 14). Bilateral salpingectomy was performed according to pelvic exploration during surgery, e.g., revealing EP combined with contralateral hydrosalpinx. Three cases with a history of bilateral fallopian tube resection showed interstitial pregnancy. Cases 12 and 18 represented the same patient who had two ectopic pregnancies. Two frozen blastocysts were transferred under the artificial cycle every time. During the first time of EP, the patient was treated conservatively; for the second time of EP, she received laparoscopic bilateral salpingectomy. Cases 22 and 23 had cornual pregnancy. Case 22 underwent ultrasound-monitored curettage. Case 23 received IVF/ICSI-ET in another hospital because of bilateral salpingectomy. She complained of lower abdominal pain for 5 days after fresh cycle embryo transfer 24 days ago. Another hospital misdiagnosed acute appendicitis before transferring the patient to the Department of Surgery of our hospital. Laparoscopic exploration indicated that the right-side corner of the uterus was ruptured, which was repaired later. Case 24 had cervical pregnancy. Due to the cessation of embryonic development, the embryonic tissue was clamped under monitoring by ultrasound and a balloon was compressed in the uterine cavity to stop bleeding. After the ectopic pregnancies were cured, four cases were transferred embryos again. Three were clinically pregnant, including that one's embryo stopped developing and one was EP treated by laparoscopic bilateral salpingectomy.

## Discussion

Compared with the EP after natural conception, the clinical manifestations of EP after embryo transfer are more complex. Understanding the complexity of EP after embryo transfer is helpful for the early recognition and treatment of EP after IVF-ET. The current study confirmed that EP after embryo transfer is very complex, with diverse manifestations, indicating that even with a pregnancy sac observed in the uterus, the pelvic cavity should be scanned thoroughly following IVF/ICSI-ET. The incidence of EP after embryo transfer was 1.29% in this study, which was slightly lower than the previously reported rate of 1.4%–5.4% [3]. For the early diagnosis of EP after natural conception, we can make a diagnosis according to the clinical symptoms of amenorrhea, abdominal pain, and vaginal bleeding, as well as doubly increased hCG and the performance of ultrasound. The clinical manifestations of EP after embryo transfer included vaginal bleeding and lower abdominal pain, in agreement with previous findings [7]. Moreover, natural conception is mainly based on the implantation of a single embryo, which rarely occurs in both intrauterine and extrauterine pregnancies or two extrauterine pregnancies at the same time. EP is more complex after embryo transfer than natural conception. Serum hCG monitoring (>2,000 IU/L) combined with transvaginal ultrasound is a reliable method for diagnosing EP [8]. Due to the transplantation of 2 embryos, compounded with luteal support, the increasing trend of hCG may not be able to detect EP. Early hCG measurements, such as day 5 post-blastocyst transfer, may predict pregnancy outcomes, distinguishing ongoing pregnancies from failing ones, including EP [9]. At the same time, because two embryos were often transferred to the intrauterine cavity, these two embryos may be implanted in different parts. Therefore, there were cases in which intrauterine pregnancy and extrauterine pregnancy occurred simultaneously, or there were two parts of extrauterine pregnancy at the same time. All of these made the diagnosis of EP after embryo transfer more complicated. For Case 2, due to sexual intercourse before embryo transfer, she had intrauterine pregnancy with double chorionic and four amniotic sacs and right tubal ampullary pregnancy. So, in the follow-up after embryo transfer, it is necessary to detect the changes of hCG and comprehensive intrauterine and extrauterine ultrasound assessments.

Ultrasound assessment is an important basis for the early diagnosis of EP. It has a high accuracy for the diagnosis of EP after natural pregnancy. However, EP after embryo transfer may be misdiagnosed by ultrasound. For case 23, the right uterine cornual pregnancy was misdiagnosed as acute appendicitis after fresh cycle embryo transfer. The clinical manifestations were not typical, and the history of bilateral salpingectomy might be overlooked in the EP. The enlarged ovaries after controlled ovarian hyperstimulation and blood bodies formed after puncture made it difficult to identify the EP mass. In this study, two patients were diagnosed with heterotopic pregnancy according to clinical manifestations and ultrasound abnormalities. But curettage confirmed that they had intrauterine pregnancy.

The desire of patients for intrauterine pregnancy also increases the difficulty of early diagnosis of EP after embryo transfer. Patients undergoing embryo transfer expected intrauterine pregnancy, and a diagnosis of EP is difficult to accept. They asked for continuous observation and declined surgery. Although EP could be treated conservatively, it might make sense to perform an active surgical evaluation of patients after embryo transfer. It was reported that laparoscopic surgery to assess pelvic conditions after IVF-ET failure helped increase the rate of pregnancy in the IVF-ET cycle [10].

From an etiological perspective, the pathogenesis of EP after embryo transfer is partly the same as that after natural pregnancy. Pelvic inflammation, endometriosis, a history of EP, a history of tubal surgery, and uterine abnormalities are risk factors for EP [11]. In the present study, the majority of ectopic pregnancies had fallopian tube involvement. Previous findings indicate that more than 90% of ectopic pregnancies occur in the fallopian tube and are associated with abnormal fallopian tubes and uterine anomalies. Inflammation of the fallopian tubes may alter their environment and transport function; meanwhile, uterine abnormalities can affect endometrial receptivity and increase uterine contractions [12]. In addition, smoking and advanced maternal age may also increase the incidence of EP [13] although other authors found no relationship between maternal age and EP risk [14].

In addition, there are different causes for EP to occur after ARTs and natural pregnancy. The occurrence of EP after embryo transfer may be related to the ART itself [11]. First of all, it may be related to superovulation. During ovarian hyper-stimulation, excessive ovarian response, IVF (as opposed to ICSI), and an GnRH (Gonadotropin releasing hormone) agonist trigger may be risk factors for EP [15]. Elevated estrogen and progesterone during superovulation may also increase the risk of developing EP [16]. Furthermore, it is known that the incidence of EP in frozen cycle embryo transfer is lower than that of fresh cycle [17]. However, whether the superovulation regimen increases the incidence of EP remains controversial. In this study, more EPs after frozen-cycle embryo transfer were found. This was because of a higher number of frozen cycles than fresh cycles in embryo transfers. Secondly, embryo factors may also be involved in EP. In theory, blastocyst stage embryo transfer is more synchronous with the endometrium, and the incidence of EP after blastocyst-stage embryo transfer is lower than that found with the cleavage stage. However, a study reported no statistically significant difference in the incidence of EP between blastocyst- and cleavage-stage embryo transfer [18]. Another report demonstrated that a single blastocyst transfer could reduce the incidence of EP [19]. In this study, there were 15 of 20 patients who were transferred the cleavage-stage embryo. There were more patients who were transferred cleavage-stage embryos than those with blastocyst-stage embryos in our center. Thirdly, EP may be related to embryo-transfer procedures. Stimulation of the uterus during embryo transfer could cause uterine contraction, and intimal peristalsis increases the movement of the embryo in the uterine cavity. Meanwhile, the graft cannula is too deep, with large amounts of fluid, which may increase the risk of EP. Finally, excessive pushing of the embryo may increase its possibility of moving to the fallopian tube, leading to EP [11].

The treatment of EP should be determined according to the specific site and clinical conditions. Generally speaking, surgery is the main treatment, such as fallopian tube resection. In addition, for patients who do not need surgery or are not suitable for surgery, conservative treatment such as drug therapy is also an important treatment, such as MTX (Methotrexate), mifepristone, etc. Doctors should pay attention to the following points. First, doctors should fully consult with the patient, explain to them the treatment procedure thoroughly, and then prepare for the subsequent embryo transfer. Secondly, after the treatment of EP, hCG should be monitored until normal levels are reached. In case the decline of hCG level is not satisfactory, the patient should be assessed for potential multiple ectopic pregnancies. In EP involving the fallopian tube, surgical methods include fallopian tube fenestration and resection. A randomized controlled trial (RCT) found that the rates of EP recurrence and intrauterine pregnancy are similar after two surgical procedures [20]. To avoid EP recurrence, patients with fallopian tube resection should be considered for close monitoring. Similarly, the possibility of EP after bilateral salpingectomy should not be ignored.

This study had limitations. First, all EP patients came from one tertiary hospital. There may have been a bias of sample selection. In addition, the retrospective design has inherent shortcomings. Finally, the sample size was relatively small. Statistical analysis of large samples may remove confounding factors, such as previous EP history.

## Conclusion

This study assessed 24 patients with EP after embryo transfer. Our findings confirmed that EP after embryo transfer, which mainly involves the fallopian tube, was very complex with diverse manifestations and might be misdiagnosed easily. Therefore, even with the pregnancy sac observed in the uterus, the pelvic cavity should be scanned thoroughly after IVF/ICSI-ET. This study reminded us that although we had a lot of experience in EP after natural conception, we should pay more attention to the difference between EP after embryo transfer and EP after natural conception to avoid misdiagnosis.
